# The Association between Social Support and Incident Dementia: A 10-Year Follow-Up Study in Japan

**DOI:** 10.3390/ijerph16020239

**Published:** 2019-01-16

**Authors:** Chiyoe Murata, Tami Saito, Masashige Saito, Katsunori Kondo

**Affiliations:** 1 National Center for Geriatrics and Gerontology, 7-430 Morioka, Obu 474-8511, Japan; t-saito@ncgg.go.jp (T.S.); kkondo@chiba-u.jp (K.K.); 2 Department of Social Welfare, Nihon Fukushi University, Okuda, Chita-gun, Mihamacho 470-3295, Japan; masa-s@n-fukushi.ac.jp; 3 Center for Well-being and Society, Nihon Fukushi University, Aichi 460-0012, Japan; 4 Center for Preventive Medical Sciences, Chiba University, 1-8-1 Inohana, Chuo-ku, Chiba 260-8670, Japan

**Keywords:** social support, dementia, longitudinal study

## Abstract

Social support is important for the health of elderly populations. However, its longitudinal effect on incident dementia is unclear. We used the Aichi Gerontological Evaluation Study (AGES) project data to investigate the longitudinal effect of social support on dementia onset. Functionally independent older people at baseline (*n* = 14,088) in 10 municipalities were followed from 2003 to 2013 using National Long-term Care Insurance System data. Social support was assessed by the following support sources: co-residing family, family or relatives living apart, and friends or neighbors. Cumulative incidence of dementia was 14.6% and 18.7% for men and women, respectively. Cox proportional hazard models were employed by gender to investigate the association between social support and dementia onset adjusting for age, health status, health behaviors, subjective cognitive complaints, depression, and other socioeconomic factors. Gender differences were observed in the association between social support and incident dementia. Support from co-residing family members was protective among men, whereas among women, no effect of social support on dementia was observed. Among other social factors, community engagement was protective for women, while for men, being married was associated with lower incidence of dementia. The association between social support and dementia seems to differ by gender. When we design programs to promote social interactions among the elderly, we need to take into account such gender differences.

## 1. Introduction

The effects of social relationships and social support are well documented in a large number of studies. Social networks or social support exchanges in supportive social relationships are related to better health. For example, those with more social networks have a lower risk of mortality [1], morbidity [2], functional disabilities [3,4,5], and cognitive impairment [6]. When people interact, something is exchanged, namely, social support. In other words, social support is embedded in social relationships [1]. Studies have also reported the association between social support and cognition. Seeman et al. [7] reported that emotional support was a significant predictor of cognitive functioning at the 7.5-year follow-up, using MacArthur Studies of Successful Aging data. Another 12-year follow-up study [8] demonstrated that baseline exposure to emotional support was independently associated with a better Mini-Mental State Examination (MMSE) score at follow-up. According to a systematic review of 19 longitudinal studies that investigated the association between social relationship factors and incident dementia, not network size per se but lack of social interaction seems to be associated with incident dementia [6].

However, given the nature of social support, cultural or gender factors cannot be ignored when assessing its effect on health. A study in the United States [9] demonstrated that gender moderates the relationship between social support and cognition; higher levels of emotional support are associated with better cognition only among females. Support sources are also important factors to consider. In general, studies in Western nations have reported that support from a wide variety of sources, including friends or neighbors, can be beneficial for the health of elderly populations [1]. On the contrary, studies in the East have reported the relative importance of family support. A cross-sectional study in China [10] demonstrated that family support is the strongest predictor of cognitive function among older persons, whereas support from friends was not. Cheng et al. [11] suggested, based on their conclusions in another cross-sectional study in China, that interactions with friends are less well-being enhancing in Asian societies compared with their Western counterparts. The authors speculated that, in societies where social harmony and reciprocity are valued, like those across Asia, the cost of seeking help from others, especially from non-family members, might be higher compared with more individualistic societies. Fiori et al. suggested that the relative importance of filial duty in Asian cultures might also explain the result [12]. Thus, the association between social support and cognition might be different depending on support sources in Asian societies such as China, Korea, and Japan compared with their non-Asian counterparts.

However, as far as we know, few studies have examined the longitudinal effect of social support on dementia in Asia. Only one study in Taiwan reported that, along with other risk factors such as physical activity or depression, social support is related to late life cognitive decline [13]. Saito et al.’s study demonstrated the relative importance of diverse social networks in preventing dementia. However, the study did not consider sources of support. In addition, gender was treated as a covariate and gender differences were unclear [14].

The purpose of this study is to assess the effect of social support on incident dementia, taking into consideration support sources by gender. Our hypothesis was that the protective effect of social support on dementia differs by source and gender, and that the effect of family support would be stronger compared with non-family support.

## 2. Materials and Methods

The present study is part of the Aichi Gerontological Evaluation Study (AGES) project [15]. The AGES project is a community-based prospective cohort study in Japan in which investigators evaluate factors associated with incident functional disability or dementia among noninstitutionalized older people aged 65 years old or above. In the baseline year of 2003, questionnaires were sent to a random sampling of community-living older adults aged 65 years or older in six large municipalities and a complete census of four small cities. The response rate was 52.1%. Detailed descriptions of the project and questions on the survey have been published [16]. After excluding those with incomplete data on sex and age, 15,313 people (7381 men and 7932 women) were introduced in the cohort and followed for about 10 years from 1 November 2003, to 28 March 2013. We obtained information regarding incident functional disability, dementia, death, and relocation of participants (e.g., moving out of the study area) from the Long-Term Care Insurance (LTCI) system database maintained by municipalities. For the analyses, we eliminated those with limitations in basic activities of daily living, such as using the toilet, bathing, or transferring at the baseline year of 2003. This procedure left 14,088 people, or 92% of the total sample of this cohort. Study participants were comparable to entire older Japanese populations in terms of age and sex [15].

### 2.1. Brief Description of Japan’s LTCI System

Japan’s LTCI system is a government-operated national insurance system for long-term care and was introduced in April 2000 to entitle every Japanese person aged 65 years and older with functional limitations or dementia to care in basic activities of daily living [17]. In this system, certification of long-term care needs is based on an evaluation of each applicant’s degree of physical and mental disability, determined by a home-visit interview and a diagnosis from a primary care physician. A municipality certification committee determines the eligibility for receiving services [17]. As receipt of benefits under this LTCI system is on an application basis, some people do not receive benefits under the system for various reasons, such as the availability of family members to provide care or financial burden (a 10% coinsurance is required to use services under the LTCI). To minimize such bias, we asked about basic activities of daily living, such as using the toilet, bathing, or transferring, in the 2003 survey and then excluded those already functionally impaired at baseline.

### 2.2. Incident Dementia

Incident dementia was ascertained when study participants became eligible for Japan’s public LTCI system, Level II or higher, on the index for the evaluation of care needs for people with dementia. The index was developed by the Ministry of Health and Welfare, based on observations of symptoms and behaviors that cause daily life impediment and degradation of cognitive functions along with communication difficulty. This index was validated using the MMSE and Revised Hasegawa Dementia Scale (HDS-R). The correlation coefficients with each scale were −0.744 and −0.735, respectively, indicating strong correlations with clinically used instruments [18]. Insurance data were provided by insurers (municipalities) per the study agreement with the AGES project [16].

### 2.3. Social Support

To elucidate social support, we asked respondents about the five types of perceived social support with respect to persons in three social support sources: co-residing family, family/relatives living apart, and friends/neighbors. “Co-residing family” refers to spouse/partner, children, or others living in the same household. “Family/relatives living apart” refers to adult children, siblings, or others not living in the same household. The types of support were emotional (providing/receiving), instrumental (providing/receiving), and appraisal (receiving). The act of “listening to concerns and complaints” was regarded as emotional support and that of “looking after when sick in bed for a few days” as instrumental support. Appraisal support was elicited by asking, “Do you have someone who acknowledges your existence and value?” These types of supports were often used in previous research studies [1,16]. Answering categories for these social support variables were coded dichotomously as “support available” (coded as 1) and “no support available” (coded as 0).

### 2.4. Covariates

Age and health status are important confounders when assessing the relationship between social support and health [1,19]. Number of illnesses was ascertained by the question, “Are you currently receiving any medical treatment, and if so, for which illnesses?” For analyses, we calculated the number of illnesses, treated as a continuous variable. We adjusted for depression as well, as depression coexists with or predicts dementia [20]. Depression was assessed by the 15-item geriatric depression scale (GDS-15), an instrument to screen depression among community-living older persons [21]. The score was transformed into a dichotomous variable (<5: no depression; 5+: depression).

Subjective cognitive complaints predict dementia among the elderly [22]. Thus, we asked respondents the following three questions: “Do you often get into trouble when you leave your belongings behind somewhere?”; “Do you often get times or places confused?”; and “Do you often forget things that happened recently (e.g., what you had for breakfast)?” The possible highest score was three, suggesting more cognitive impairments. As living arrangement is strongly associated with health [19], we adjusted for its effect as well by stratifying those living alone and those living with someone else. We adjusted for marital status as well, since a small number (*n* = 291) of respondents did not live with their spouse even while they were married, probably owing to hospitalization or institutionalization of their spouse. Engagement in community activities was ascertained by asking respondents about the number of community groups they participated in, such as sports, hobby, or local associations, as such participation is protective against dementia [20]. Health behaviors such as smoking status, daily physical activity (average walking time per day), alcohol consumption, and education were also considered in the model.

### 2.5. Statistical Analyses

As studies have indicated that the association between social support and health differ by gender [1], Cox proportional hazard models were employed stratified by gender to assess the association between baseline social support and incident dementia. Those who died or moved away from the study site during the follow-up period were considered as censored cases. To test if the effects of social support from each source were independent of the influence of others, we entered social support in the model along with other covariates stratified by support source. As our main aim was to study the relative effect of social support, social support variables were aggregated to create social support scores by support source (co-residing family members, family/relatives living apart, and friends/neighbors).

Moreover, we tested which support type was the most beneficial when coming from the same support source. The correlation coefficients of social support variables were as high as 0.705, for example, between “providing instrumental support” and “receiving instrumental support” for family members living together. As such, we constructed three models stratified by support source to avoid multi-collinearity. Then, we investigated the association between each social support and dementia. We used SPSS 21.0J (SPSS, Chicago, IL, USA) for statistical analyses. A *p*-value of less than 0.10 was considered marginally significant, and a *p*-value less than 0.05 was considered statistically significant.

### 2.6. Ethical Issues

The study protocol and informed consent procedure were approved by the Nihon Fukushi University Ethics Committee (#10-05). The study was conducted in compliance with the fifth revision of the Declaration of Helsinki.

## 3. Results

Table 1 shows baseline characteristics by gender. During the 10-year follow-up, 14.6% of men and 18.7% of women developed dementia. Men were slightly younger and participated in more community groups but smoked and drank more alcoholic beverages. More women lived alone and had more cognitive complaints than did men. In addition, women were significantly more depressed and had more illnesses.

Table 2 describes social support by type and source. As for social support, men exchanged more support with co-residing family members, whereas women had more ties with family/relatives living apart or friends/neighbors. When subdivided by sources, men exchanged more emotional and instrumental support with their co-residing family and received more appraisal support from all sources than women did. Women exchanged more emotional and instrumental support with someone outside of their own households, namely a family member/relative who lives apart or friends/neighbors. When considering the effect of support sources, men benefit more from support exchanges with their co-residing family members, as shown in Table 3. As for types of support, providing support to co-residing family was a significant protector against dementia among men, whereas among women, providing emotional support to family/relatives who live apart and receiving emotional support from friends or neighbors were protective against dementia (Table 4). Contrary to our expectations, instrumental support exchanges with friends or neighbors were risks for dementia among men. Among women, receiving emotional support from co-residing family members raised the risk of dementia, whereas providing support to a family/relative who lives apart and receiving emotional support from friends or neighbors were protective of dementia (Table 4).

## 4. Discussion

### 4.1. Support to/from Co-Residing Family

Using 10-year follow-up data, we assessed the impact of social support on incident dementia by sources (support exchange partners). Contrary to our expectations, family support was beneficial only for men. In our data, although we cannot ascertain with whom men exchanged support in the house, in the sub-analysis, 92.5% of men with support from co-residing family were married. This supports the result of Saito et al.’s study which found similar associations that, among men, support from spouse was an independent protective factor against functional disability [23]. In that study, lack of social support explained 24.4% and 15.8% of the excess risk of disability among men living alone and those living with non-spousal cohabitants, respectively. This also is in line with a review that indicated that men tend to report their spouses as confidants, whereas women do not [1,19]. Cognitive benefits of support from co-residing family members for men could also be attributed to enhanced social roles or self-efficacy by providing support to their spouse. Another reason for such difference is the difference in the nature of support they receive, in addition to women’s longer life span. Men tend to provide more practical help such as bringing them to hospital, whereas women tend to provide more essential help in daily living, such as cooking or cleaning the house and giving emotional assurance. This means that men with care needs might live without applying for insurance benefits as long as their spouse can provide essential care. This might lead to delayed diagnosis of dementia among men.

Among women, receiving emotional support from co-residing family members was a risk for dementia. Goldzweig et al. reported that women often feel guilty when they cannot perform household chores [24]. Such feelings of guilt might undermine their self-image and lead to poorer mental health, a risk factor for dementia. A cross sectional study in the United States demonstrated a negative association between social support and cognition, in which, among older persons, greater social support was associated with poorer nonverbal memory and response inhibition, suggesting their care needs [25]. Marital benefits among men might partly explain the result. A study reported that married men reported significantly higher levels of spouse support compared with married women [24].

### 4.2. Support to/from Family Living Apart

In our study, social support from family or relatives living apart had no significant effect on cognition in both genders, except for the provision of emotional support among women. A study in China demonstrated the importance of extended family for the well-being of older persons, especially when support from immediate kin was not available [12]. In Japan, more adult children live separately from their parents once they get married. When needs arise, they take care of their older parents. Thus, the availability of support from family or relatives living apart might indicate the actual care needs among older parents.

Another explanation is that the nature of support might be important for older persons’ health. A review of studies reported that receiving emotional support from kin members raised the well-being of older persons, whereas receiving instrumental support did not when the quality of relationships was low [1]. Because kin relationships are not easy to terminate even if the quality is poor, this might lead to feelings of guilt or dependency and even reduced autonomy. A study using the English Longitudinal Study of Ageing (ELSA) data with a 10-year follow-up period reported that positive social support from children reduced the risk of dementia, whereas negative support increased the risk among persons aged 50 years and over [26]. Although we cannot assess such an effect in our study due to the lack of related variables, quality rather than quantity of social relationships might be important for cognitive health.

### 4.3. Support to/from Friends or Neighbors

Among men, instrumental support exchange with friends or neighbors was a risk for dementia. Psychological factors in part explain this result. Studies have reported that social relations lacking in mutuality often undermine the self-image of the support recipient. Receiving support from non-family members could be considered a shame or threat to men’s masculinity. Goldzweig et al. reported men’s lower ability to use social support compared with women based on their observations that, despite receiving more support, men tend to report more psychological distress in the face of illness [24]. Also, men are less likely to seek help for physical and psychological problems [27].

Another possibility is gender differences in social relations. In Japan, non-family support is mainly provided by same gender individuals [28]. Male friends are more likely to advise friends to receive treatment with deteriorated cognition, while female friends tend to provide essential help and emotional assurance, so that those in early stages of dementia could continue living in the community. In the sub-analyses, we assessed the characteristics of men who exchanged instrumental support with friends or neighbors (*n* = 450). They were more likely to be married (78.9%) and living with someone else (86.4%). In addition, more than 90% of them reported that they exchanged support with co-residing family. Thus, the likelihood of dementia among men with instrumental support from their friends or neighbors could be partially explained by their deteriorated cognition that could be easily noticed by others.

Among social factors, community engagement was more strongly associated with lower incidence of dementia among women, while for men, being married was a stronger protector against dementia (data shown upon request). This is in line with studies in Western nations, in which men received emotional support mainly from their spouses, whereas women received more support from their friends and relatives or children [6,29]. A previous study with the same dataset reported a similar association, in which broader social networks were protective against incident dementia among women [14]. This may also be explained by the replacement function of social networks. Women often live longer than men and cannot rely on their husbands when needs arise. In fact, in our previously reported study finding, women living alone had more contacts with outside non-family members [30]. Also, relationships with non-kin members, such as friends or neighbors, are mostly voluntary and based on mutual trust and love, meaning more positive support [31]. In addition, to interact with non-family persons, one must go out of one’s own home, which could lead to receiving more physical and cognitive stimuli in daily life.

### 4.4. Strengths and Limitations

This study’s major strength is the use of large insurance data, with few missing cases, that were maintained by municipalities. The present study adds several new findings to those of earlier studies. First, a longitudinal protective effect of social support on dementia was suggested. Second, different effects of social support by type and source were observed. Third, gender differences were also observed in the association.

One limitation of this study is the use of a proxy measure for incident dementia. As mentioned in the introduction, a certain percentage of people do not apply for insurance benefits for various reasons. Although we eliminated those with impairment in basic activities in daily living and adjusted for baseline cognitive complaints, people with undiagnosed dementia might have been included in the study population. In addition, the presence of a spouse might delay the diagnosis of dementia among men, due to spousal support in daily living, and lead to misclassification of cases. Also, the representativeness of the population might be an issue. When we assessed the tendency of non-responders, those aged 85 years and over and those with lower income were less likely to respond [16], meaning that the risk of incident dementia might be underestimated in this study, since incident dementia is higher among those groups [16].

Another limitation is that only baseline social support was measured. As people get older, their life space is more likely to diminish and be confined to a narrower network of people. Also, the effect of social support might diminish over time. To consider such a possibility, we repeated sensitivity analyses with shorter follow-up periods. However, hazard ratios did not change much (data available upon request). Studies have reported that, despite the number of social ties diminishing over time among older persons, the amount of emotional and instrumental support did not [7], indicating a relatively stable effect of social support. Nonetheless, studies with repeated measurements that can consider changes in social support might be desirable.

## 5. Conclusions

Gender differences were observed in the association between social support and incident dementia. Support from co-residing family members was protective among men, whereas among women, no significant effect of social support on dementia was observed. Considering the gender differences in the association between social support and dementia and the possibility of misclassification of cases, the use of objective diagnosis data might be necessary. Also, given the fact that the number of older people living alone is increasing [2], there is a greater need to investigate the effect of promoting social ties with outside family members. As men benefit more from support from co-residing family members, different strategies might be needed in designing intervention programs for men. Deteriorating cognitive or physical functions might undermine their self-image, leading to poor health; this occurs especially among men who want to be strong and do not want to show their weakness to others [24]. In our study, appraisal support from someone outside of their own homes was related to a lower hazard for dementia, although it was not significant. If support is provided with respect, or if recipients are given more of a chance to give back support, this might preserve autonomy and mitigate the negative effect of receiving support, especially among men. Such support could enhance their self-esteem and compensate for the threat of loss of autonomy from receiving support.

## Figures and Tables

**Table 1:** Baseline characteristics by gender.

	Mean ± SD or *n* (%)				
	Men (*n* = 6906)		Women (*n* = 7182)		*p*-Value ^1^
Age in years (65–99)	72.3	±5.63	73.1	±6.10	<0.001
# of illnesses (0–19)	1.58	±1.36	1.71	±1.46	<0.001
Geriatric Depression Scale (5+)	2458	35.6	3098	43.1	<0.001
Subjective cognitive complaint (0–3)	0.70	±0.93	0.78	±0.92	<0.001
Smoker (Yes)	1596	(23.1)	180	(2.5)	<0.001
Alcohol consumption (Yes)	3936	(57.0)	881	(12.3)	<0.001
Sedentary (<30 min walk a day)	2203	(31.9)	2289	(31.9)	n.s.
Married (Yes)	6104	(88.4)	4002	(55.7)	<0.001
Education (<10 years)	3802	(55.6)	4502	(63.5)	<0.001
Living alone (%)	287	(4.2)	1098	(15.3)	<0.001
Engagement in community activities ^2^ (0–8)	1.50	±1.43	1.36	±1.32	<0.001
Incident dementia	1008	14.6	1343	18.7	<0.001

^1^*p*-values are for gender differences. *p*-values were calculated by chi-squared test for categorical variables and *t*-test for continuous variables. n.s. not significant. ^2^ Engagement in community activities was the sum of the number of community groups, such as sports, hobby, or local associations that the individuals participated in.

**Table 2:** Description of social support by type and source of support.

	Co-Residing Family	Family/Relatives Living Apart	Friends/Neighbors
Men (*n* = 6906)			
Receiving emotional support	4797 (69.5)	1665 (24.1)	1361 (19.7)
Providing emotional support	3922 (56.8)	1916 (27.7)	1970 (28.5)
Receiving instrumental support	5938 (86.0)	1593 (23.1)	193 (2.8)
Providing instrumental support	5628 (81.5)	1822 (26.4)	414 (6.0)
Receiving appraisal support	5490 (79.5)	2486 (36.0)	1576 (22.8)
Women (*n* = 7182)			
Receiving emotional support	3849 (53.6)	3068 (42.7)	2558 (35.6)
Providing emotional support	2411 (33.6)	2624 (36.5)	3417 (47.6)
Receiving instrumental support	4870 (67.8)	2644 (36.8)	428 (6.0)
Providing instrumental support	4770 (66.4)	2860 (39.8)	962 (13.4)
Receiving appraisal support	4669 (65.0)	3206 (44.6)	1672 (23.3)

Figures in the table are numbers. Percentages are in parenthesis.

**Table 3:** Social support and incident dementia by Cox proportional hazard models.

	Men (*n* = 6906)		Women (*n* = 7182)	
	HR	95% CI	HR	95% CI
Social support				
Co-residing family (0–5)	0.95	(0.91–0.99) *	1.00	(0.97–1.04)
Family/relatives living apart (0–5)	1.03	(0.99–1.08)	1.00	(0.96–1.03)
Friends/neighbors (0–5)	0.95	(0.89–1.02)	0.96	(0.92–1.01)

* *p* < 0.05. Social support is the sum of the five types of social support (providing/receiving emotional support, providing/receiving instrumental support, and receiving appraisal support). Social support to/from each source was entered into the model simultaneously to mutually adjust for each effect. Covariates (age, number of illnesses, geriatric depression scale, subjective cognitive complaints, smoking, walking, alcohol consumption, education, and community engagement) were also entered into the model.

**Table 4:** Social support by source and type and incident dementia.

	Men		Women	
	HR	95% CI	HR	95% CI
Co-residing family				
Receiving emotional support	0.92	(0.79–1.06)	1.14	(1.00–1.30) *
Providing emotional support	0.83	(0.72–0.95) **	1.07	(0.94–1.22)
Receiving instrumental support	0.87	(0.71–1.05)	0.94	(0.81–1.09)
Providing instrumental support	0.80	(0.68-0.95) *	0.89	(0.77–1.02) †
Receiving appraisal support	1.00	(0.84–1.18)	0.95	(0.82–1.09)
Family/relatives living apart				
Receiving emotional support	1.13	(0.98–1.31)	0.98	(0.87–1.11)
Providing emotional support	1.11	(0.96–1.29)	0.87	(0.76–0.98) *
Receiving instrumental support	1.06	(0.91–1.24)	1.07	(0.94–1.21)
Providing instrumental support	1.02	(0.88–1.19)	0.97	(0.85–1.10)
Receiving appraisal support	0.94	(0.82–1.08)	0.97	(0.86–1.10)
Friends/neighbors				
Receiving emotional support	0.82	(0.68–0.98) *	0.85	(0.74–0.97) *
Providing emotional support	0.85	(0.72–0.99) *	0.92	(0.81–1.04)
Receiving instrumental support	1.57	(1.14–2.18) **	1.08	(0.83–1.41)
Providing instrumental support	1.33	(1.03–1.71) *	0.90	(0.74–1.10)
Receiving appraisal support	0.87	(0.73–1.03)	0.96	(0.83–1.12)

† *p* < 0.10; * *p* < 0.05; ** *p* < 0.01. Cox hazard models were employed stratified by support sources. Presence or absence of each social support type was entered separately for each support source to avoid multi-collinearity. All covariates (age, number of illnesses, subjective cognitive complaints, depression, health behaviors, living arrangement, marital status, education, and engagement in community activities) were also entered into the model.
